# Targeting arginase-1 exerts antitumor effects in multiple myeloma and mitigates bortezomib-induced cardiotoxicity

**DOI:** 10.1038/s41598-022-24137-1

**Published:** 2022-11-16

**Authors:** Kavita Ramji, Tomasz M. Grzywa, Anna Sosnowska, Aleksandra Paterek, Marta Okninska, Zofia Pilch, Joanna Barankiewicz, Filip Garbicz, Katarzyna Borg, Urszula Bany-Laszewicz, Abdesamad Zerrouqi, Beata Pyrzynska, Anna Rodziewicz-Lurzynska, Diana Papiernik, Piotr Sklepkiewicz, Hanna Kedzierska, Adam Staruch, Radoslaw Sadowski, Olga Ciepiela, Ewa Lech-Maranda, Przemyslaw Juszczynski, Urszula Mackiewicz, Michal Maczewski, Dominika Nowis, Jakub Golab

**Affiliations:** 1 Department of Immunology, Medical University of Warsaw, 5 Nielubowicza Str., 02-097 Warsaw, Poland; 2 Laboratory of Experimental Medicine, Medical University of Warsaw, 5 Nielubowicza Str., 02-097 Warsaw, Poland; 3 Department of Clinical Physiology, Centre of Postgraduate Medical Education, 99/103 Marymoncka Str., 01-813 Warsaw, Poland; 4 Department of Hematology, Institute of Hematology and Transfusion Medicine, 14 Indiry Gandhi Str., 02-776 Warsaw, Poland; 5 Department of Experimental Hematology, Institute of Hematology and Transfusion Medicine, 14 Indiry Gandhi Str., 02-776 Warsaw, Poland; 6 Postgraduate School of Molecular Medicine, Medical University of Warsaw, 1B Banacha Str., 02-097 Warsaw, Poland; 7 Department of Diagnostic Hematology, Institute of Hematology and Transfusion Medicine, 14 Indiry Gandhi Str., 02-776 Warsaw, Poland; 8 Central Laboratory, University Clinical Center of Medical University of Warsaw, 1A Banacha Str., 02-097 Warsaw, Poland; 9 ExploRNA Therapeutics Ltd., 101 Zwirki I Wigury Str., 0.30, 02-089 Warsaw, Poland; 10 Department of Laboratory Medicine, Medical University of Warsaw, 1A Banacha Str, 02-097 Warsaw, Poland; 11 Centre of Preclinical Research, Medical University of Warsaw, 1B Banacha Str., 02-097 Warsaw, Poland

**Keywords:** Immunotherapy, Tumour immunology

## Abstract

Multiple myeloma (MM) remains an incurable malignancy of plasma cells despite constantly evolving therapeutic approaches including various types of immunotherapy. Increased arginase activity has been associated with potent suppression of T-cell immune responses in different types of cancer. Here, we investigated the role of arginase 1 (ARG1) in Vκ*MYC model of MM in mice. ARG1 expression in myeloid cells correlated with tumor progression and was accompanied by a systemic drop in ʟ-arginine levels. In MM-bearing mice antigen-induced proliferation of adoptively transferred T-cells was strongly suppressed and T-cell proliferation was restored by pharmacological arginase inhibition. Progression of Vκ*MYC tumors was significantly delayed in mice with myeloid-specific ARG1 deletion. Arginase inhibition effectively inhibited tumor progression although it failed to augment anti-myeloma effects of bortezomib. However, arginase inhibitor completely prevented development of bortezomib-induced cardiotoxicity in mice. Altogether, these findings indicate that arginase inhibitors could be further tested as a complementary strategy in multiple myeloma to mitigate adverse cardiac events without compromising antitumor efficacy of proteasome inhibitors.

## Introduction

Multiple myeloma (MM) is a malignancy characterized by the uncontrolled expansion of clonal plasma cells (PC) accompanied by clinical symptoms, such as bone destruction, renal injury, anemia, hypercalcemia, and paraproteinemia^1^. The disease follows a multistep process that usually starts with premalignant monoclonal gammopathy of undetermined significance (MGUS) and progresses through smoldering multiple myeloma (SMM) to active MM. Disease progression is associated with accumulation of genomic alterations and development of immune dysfunction that increase the risk of infectious complications and contributes to tumor progression^2,3^. Antitumor efficacy of MM treatment has greatly improved over the recent years, with 5-year overall survival now exceeding 50%. Despite great progress in the management of MM, active disease remains incurable. A dysfunction of innate and adaptive immunity, particularly affecting T-cell responses, indicates that development of novel approaches to enhance antitumor immunity is a rational and promising strategy.

Multiple myeloma is largely confined to the bone marrow (BM), but with time it spreads to other tissues. MM cells strongly depend on the interactions with BM microenvironment and BM stromal cells are a rich source of cytokines and growth factors (such as CXCL4, IL-18, BAFF, APRIL, VEGF) that support proliferation, confer resistance to apoptosis and exert immunoregulatory effects. Malignant plasma cells also induce BM stromal cells, including osteoblasts and myeloid cells, to produce IL-6, thereby promoting their own proliferation^4,5^. Intriguingly, IL-6 is considered to be a very strong inducer of arginase 1 (ARG1) in myeloid cells^6^. Increased ARG1 activity has been observed in patients with various malignancies, and is considered to be involved in the suppression of the immune system^7–9^. Low ʟ-arginine concentrations are associated with down-regulation of T-cell receptor (TCR) CD3ζ chain, which is a critical component of the TCR signaling pathway^10^. In this mechanism, ʟ-arginine degradation leads to an arrest in T-cell cycle progression, inhibition of IFN-γ production, and blocking of cytotoxic effects of T-cells^9^. ARG1 is mainly produced by myeloid-derived suppressor cells (MDSCs) as well as more mature myeloid cells that are highly enriched in the tumor microenvironment. T-cell functions are restored and tumor growth is inhibited upon inhibition of arginase of tumor-associated MDSCs^11–13^ or tumor-infiltrating CD11b^+^Gr-1^–^ mature myeloid cells^12^ in murine tumor models. The increased arginase activity has been shown to participate in the suppression of tumor-infiltrating lymphocytes in mouse tumor models and in patients with various types of tumors^9,12,14^. However, ARG1 activity has not been extensively studied in MM so far. Granulocytic MDSCs (G-MDSCs) were reported to produce ARG1 in MM patients, and ʟ-arginine analog (NorNOHA) has been shown to partially restore T-cell proliferation that is suppressed by myeloid cells in patients with MM^15,16^. A study in syngeneic MM model indicated that ARG1 might play a role in suppressing antitumor immune response—intraperitoneal (i.p.) administration of recombinant ARG1 into mice with NS-1 MM significantly shortened the survival of tumor-bearing animals and impaired NK cells activity^17^. Moreover, bortezomib treatment was associated with increased serum ARG1 levels in MM patients and was suggested to contribute to treatment resistance^18^. These observations prompted us to systematically investigate the role of ARG1 in MM progression in a murine Vκ*MYC model that recapitulates many features of human MM^19,20^. Additionally, considering our previous studies showing that bortezomib impairs left ventricle ejection fraction^21^ and that the treatment of mice with ARG1 inhibitor leads to increased plasma NO concentrations, we investigated whether arginase inhibitor can mitigate adverse cardiovascular effects of proteasome inhibitor in mice.

## Materials and methods

### Mice

All experiments were performed in 8–12-week-old female mice. Wild type (WT) C57BL/6 mice were obtained from the Animal House of the Medical Research Center, Polish Academy of Sciences (Warsaw, Poland). Transgenic mice: C57BL/6-Tg(TcraTcrb)1100Mjb/J (OT-I, stock #003831), B6.129S4-*Arg1*^tm1Lky^/J (YARG, stock #015857), C57BL/6-*Arg1*^tm1Pmu^/J (Arg1^flox^ stock #008817), B6.129P2-*Lyz2*^tm1(cre)Ifo^/J (LysMcre, stock #004781) were purchased from Jackson Laboratory. Mice constitutively lacking functional *Arg1* in myeloid cells (myelo Arg1 KO mice) were generated as described previously^22^. Animals were housed in controlled environmental conditions in specific-pathogen-free (SPF) (transgenic mice) or conventional (WT mice) animal facility of the Medical University of Warsaw with water and food provided ad libitum. The experiments were performed in accordance with the guidelines approved by the 1^st^ Local Ethics Committee in Warsaw (approval No. 618/2018), and in accordance with the requirements of the EU (Directive 2010/63/EU) and Polish (Dz. U. poz. 266/15.01.2015) legislation. The random allocation of mice to experimental groups was done using https://www.graphpad.com/quickcalcs/randomize1.

### Vκ*MYC MM model

C57BL/6 WT, Arg1^flox^ or myelo Arg1 KO mice were intravenously transplanted with 1 × 10^6^ Vĸ*MYC cells (a kind gift from Prof. Leif Bergsagel, Mayo Clinic College of Medicine, USA). In this syngeneic MM model the disease develops in the spleen and bone marrow. Vĸ*MYC MM model tightly recapitulates human MM features, including bone and kidney involvement^23^ as well as anti-myeloma drug sensitivity^20^. Three weeks after transplantation the development of MM was confirmed with serum protein electrophoresis (SPEP) using Sebia Hydragels and HYDRASYS analyzer as increasing ratio of fraction 6 (monoclonal protein)-to-fraction 1 (albumins) [fr6:fr1].

### Treatment

Bortezomib was purchased from Adamed, Poland, dissolved in 0.9% NaCl, and administered at 0.5 or 1 mg/kg intraperitoneally (i.p.) in total four doses beginning at week 3 after inoculation of Vĸ*MYC cells. Arginase inhibitor INCB01158 was provided by Incyte and Calithera Biosciences as per os (p.o.) formulation and was administered at a dose of 100 mg/kg via oral gavage twice daily for 10–14 days beginning week 3 after inoculation of Vĸ*MYC cells. Control mice received PBS i.p. and/or p.o.

### Serum ʟ-arginine and ʟ-ornithine evaluation

Blood from the facial veins puncture was collected into Microvette® tubes with lithium heparin (Sarstedt). Samples were centrifuged at 1000 × g for 10 min, plasma was separated and kept frozen at − 20 °C until analysis. Measurements of the plasma concentration of ʟ-arginine and ʟ-ornithine were performed by ultra-performance liquid chromatography tandem mass spectrometry (UPLC-MS/MS) method on Waters Xevo TQ-S mass spectrometer equipped with Waters Acquity UPLC chromatograph (Waters) in the Mass Spectrometry Lab at the Institute of Biochemistry and Biophysics, Polish Academy of Sciences, Warsaw, Poland.

### Immunophenotyping of mouse spleen and BM cells

At indicated time points after inoculation of Vĸ*MYC cells, spleens and BM were harvested, mashed through a 70 µm nylon mesh (Corning) into single-cell suspension and the red blood cells were lysed using ACK solution (Thermo Fisher Scientific). For detection of cell surface antigens, cells were first stained with Zombie-NIR Fixable Viability Kit (BioLegend) according to manufacturer’s instructions, blocked on ice with 5% normal rat serum in FACS buffer (PBS; 1% BSA, 0.01% NaN_3_) and then incubated for 30 min on ice with fluorochrome-conjugated antibodies (listed in Supplementary Table 1). When necessary, controls for background staining such as isotype or FMO (fluorescence minus one) controls were applied. After washing with FACS buffer, cells were immediately acquired on FACS Canto II (Becton Dickinson) instrument operated by FACSDiva v 8.0 software. For data analysis Flow Jo v7.6.5 software (Tree Star) was used^22^. Gating strategies used for identification of MM cells, myeloid cells, macrophages, DCs, monocytes/monocytic- and granulocytes/granulocytic MDSCs, and subgating strategies for Ly6C^+^ and Ly6G^+^ cells are presented in Supplementary Figs. 1–6, respectively.

### Human bone marrow and PBMCs samples

*Healthy bone marrow* Healthy human bone marrow samples were commercially obtained from Lonza Walkersville, Inc. Aspirates were withdrawn from bilateral punctures of the posterior iliac crests. Every 100 ml of bone marrow was collected into syringes containing 10 ml of heparin (Porcine Intestinal Mucosa) Sodium Injection (~ 100 units heparin per ml bone marrow). Bone marrow samples were obtained from healthy males (n = 7) and healthy non-pregnant females (n = 3) US-based donors between the ages of 23 and 45 years old. Samples were collected after obtaining permission for their use in research applications by informed consent. All donors were screened for general health and negative medical history for heart disease, kidney disease, liver disease, cancer, epilepsy, and blood or bleeding disorders. All donors had negative clinical laboratory tests for HIV-1, HIV-2, hepatitis B, and hepatitis C^9^. After collection, bone marrow aspirates were centrifuged, and red blood cells were lysed using ACK (Ammonium-Chloride-Potassium) Lysing Buffer (ThermoFisher Scientific) according to the manufacturer’s protocols. The remaining stromal bone marrow cells were washed with PBS twice and used for further analysis.

*Bone marrow from MM patients* The human myeloid cells were obtained from bone marrow biopsy aspirates of MM patients as part of a routine diagnostic procedure. The selection of CD138-negative cells was performed by EasySep™ Human CD138 Positive Selection Kit II (STEMCELL Technologies) according to the manufacturer’s protocol. The heparinized bone marrow samples were centrifugated, washed with phosphate-buffered saline and lysed with 1X EasySep RBC Lysis Buffer for erythrocytes elimination. Next, the cells were incubated with antibodies, microbeads and magnetically isolated for CD138-positive fraction (plasmocytes). The flow-through of the CD138-negative fraction was washed two times with phosphate-buffered saline and used for further analyses. The written informed consent to use bone marrow samples for routine diagnostic and experimental studies was given by all MM patients.

*Peripheral blood mononuclear cells (PBMC)* Were isolated from buffy coats commercially obtained from the Regional Blood Centre in Warsaw, Poland. PBMC were isolated by density-gradient centrifugation method using Lymphoprep™ (STEMCELL Technologies), according to the manufacturer’s protocols. All donors were males between the ages of 18 and 45 years old. Donors were screened for general health and qualified by the physician for blood donation. All donors had negative clinical laboratory tests for HIV-1, HIV-2, hepatitis B, and hepatitis C and hematology values within normal ranges^9^.

### Evaluation of ARG1 levels and immunophenotyping of human BM cells

Flow cytometry was performed on FACSCanto II (BD Biosciences) operated by FACSDiva v 8.0 software. For cell surface staining, cells were stained with Zombie NIR™ Fixable Viability Kit (BioLegend), blocked on ice with 5% normal rat serum in FACS buffer (PBS; 1% BSA, 0.01% sodium azide) for 10 min, and then incubated for 30 min on ice with fluorochrome-labeled antibodies. The list of antibodies used for the staining is presented in Supplementary Table 2. When necessary, controls for background staining such as isotype or FMO (fluorescence minus one) controls were applied. After washing in FACS buffer, cells were immediately analyzed.

For intracellular staining, membrane-stained cells were fixed using Fixation Buffer for 30 min at RT, followed by a wash with permeabilization buffer, and staining with an antibody diluted in permeabilization buffer for 30 min at RT according to the manufacturer’s protocols (Intracellular Fixation & Permeabilization Buffer Set, eBioscience). After washing in FACS buffer, cells were immediately analyzed as described^9,22^.

### In vivo T cells proliferation assay

In vivo T cells proliferation assays were done during the 6th week after Vĸ*MYC cells inoculation. Ovalbumin (OVA)-derived peptide 257–264 (SIINFEKL)-specific CD8^+^ T-cells were isolated from the spleen and lymph nodes of C57BL/6-Tg(TcraTcrb) 1100Mjb/J (OT-I) mice using EasySep™ Mouse CD8^+^ T Cell Isolation Kit (Stem Cell Technologies, according to the manufacturer’s manual) and labeled with CTV (Thermo Fisher Scientific) for 20 min at 37 °C at a final concentration of 5 μM. Next, 1.5–2 × 10^6^ cells in 100 μl of PBS were immediately transferred into the caudal vein of host Vĸ*MYC-bearing or control C57BL/6 mice. The next day, host mice were challenged with 10 μg of full-length OVA protein (grade VII, Sigma-Aldrich) injected intravenously in 100 μl of PBS. The negative control (OT-I no OVA) group did not receive OVA nor was inoculated with Vĸ*MYC cells. Where indicated, INCB01158 was administered twice daily by oral gavage at a dose of 100 mg/kg, starting on the 3^rd^ week post inoculation of the Vĸ*MYC cells until the end of the experiment. On day 3 post immunization, spleens were harvested, and mashed through a 70 µm nylon strainer followed by the RBC lysis using ACK Lysing Buffer (ThermoFisher Scientific). Next, splenocytes were stained with anti-CD45.2, anti-CD3e and anti-CD8 antibodies (Supplementary Table 1) and analyzed for proliferation in flow cytometry (FACSCanto II, BD Biosciences). Percentages of proliferating cells were calculated using the FlowJo Software v10.6.1 (Tree Star).

### In vitro proliferation of murine T cells co-cultured with Vĸ*MYC-bearing mice spleen-derived CD11b^+^ cells

Spleens of control and Vĸ*MYC-bearing mice were harvested, mashed through a 70 µm nylon mesh (Corning) followed by 10 min DNase I (Sigma Aldrich, final concentration of 100 µg/ml) digestion at RT. Next, the CD11b^+^ cells were isolated using EasySep™ Mouse CD11b Positive Selection Kit II (Stem Cell Technologies, according to the manufacturer’s manual. Healthy mice CD8^+^ T cells were isolated from the spleens using EasySep™ Mouse CD8^+^ T Cell Isolation Kit (Stem Cell Technologies) and stained with Cell Trace Violet (CTV) (Thermo Fisher Scientific) for 20 min at 37 °C at a final concentration of 5 μM. Next, the co-cultures of CD11b^+^ and CTV-labelled CD8^+^ T cells at indicated ratios were established in 96-well round-bottom plates in RPMI medium (Thermo Fisher Scientific) supplemented with 10% (v/v) FBS (Thermo Fisher Scientific), 2 mM glutamine, 1% (v/v) penicillin/streptomycin, 1% (v/v) MEM non-essential amino acids solution (Thermo Fisher Scientific), 50 μM 2-Mercaptoethanol (Thermo Fisher Scientific) and 30 U/ml of recombinant human IL-2 (Peprotech). To trigger proliferation, T-cells were stimulated with Dynabeads Mouse/Human T-Activator CD3/CD28 (ratio 1:1, Thermo Fisher Scientific). INCB01158 arginase inhibitor was added at a final 1 μM concentration and the cells were co-cultured for 72 h at 37 °C in 5% CO_2_. Next, the cells were harvested, stained with Zombie NIR™ Fixable Viability Kit (BioLegend), corresponding anti-CD3 and anti-CD8 antibodies (Supplementary Table 1), and analyzed in flow cytometry (FACSCanto II, BD Biosciences). The gate for proliferating cells was set based on the unstimulated controls. Cell autofluorescence was determined using CTV-unstained controls. Percentages of proliferating cells were calculated using the FlowJo Software v10.6.1 (Tree Star).

### Human T cells proliferation assays

Human T cells were isolated from PBMC using EasySep™ Human CD4^+^ or CD8^+^ T Cell Isolation Kit (STEMCELL Technologies) according to the manufacturer’s protocols. For cell proliferation assay, T cells were labeled with Cell Trace Violet (CTV) dye (Thermo Fisher Scientific) for 20 min at 37 °C at a final concentration of 2.5 μM in PBS. Next, the labeled T cells were plated in round-bottomed 96-well plates (2 × 10^4^ cells per well) in ʟ-arginine-free RPMI medium (SILAC RPMI medium, Thermo Fisher Scientific) supplemented with 10% (v/v) dialyzed FBS (Thermo Fisher Scientific), 2 mM glutamine, 100 U/ml penicillin, 100 μg/ml streptomycin, 1% (v/v) MEM non-essential amino acids solution (Thermo Fisher Scientific), 50 μM 2-mercaptoethanol (Thermo Fisher Scientific), and 150 µM ʟ-arginine and 40 mg/l ʟ-lysine (Sigma-Aldrich). Proliferation was triggered by the stimulation with Dynabeads Human T-Activator CD3/CD28 (ratio 1:2, Thermo Fisher Scientific). T-cells were co-cultured with BM stromal cells in T cells to BM cells ratios 1:0.3, 1:1, 1:3, 1:10^9,22^.

### Nitric oxide (NO) measurement in plasma

Blood was collected by cardiac puncture and centrifuged at 2000 × g for 8 min. Next, plasma was transferred to a new tube and frozen at − 80 °C. Nitrite concentration in the collected samples was determined by a gas phase chemiluminescence reaction of NO with ozone using a Nitric Oxide Analyzer (NOA, Sievers Instruments). In this method, nitrate is reduced to NO gas in the purge vessels of the analyzer by potassium iodide in glacial acetic acid^24^.

### Echocardiography imaging in mice

Transthoracic echocardiography was performed using E-cube 15 Platinum (Alpinion Medical Systems) with 17 MHz linear transducer with mice being lightly sedated by isoflurane to maintain the heart rate > 400 bpm. After sedation mice were placed on the heating pad to sustain proper body temperature. Images of the parasternal short-axis view (SAX) at the papillary muscle level, parasternal long axis view (PLAX) and the apical 4-chamber view (4CH) was recorded with careful attention to obtain a high frame rate. LV end-diastolic (LVEDA) and end-systolic (LVESA) areas were determined from the parasternal long-axis view. LV end-diastolic and end-systolic areas. Ejection fraction (LVEF), as a marker of LV systolic function, was calculated from Simpson method. In the SAX view, M-mode for left ventricle was performed for assessment LV morphology: thickness of LV anterior and posterior walls at end cardiac diastole (LVAWd, LVPWd), LV dimensions at end cardiac diastole and systole (LVIDd, LVIDs) and LV fractional shortening (LVFS). LV diastolic function was assessed by pulsed Doppler of the mitral inflow, with use of parameters as E/A ratio, deceleration time (DecT), isovolumic contraction time (IVCT), isovolumic relaxation time (IVRT), and ejection time (ET). All measurements were obtained by one observer blinded to the study groups.

### Statistical analysis

Data are shown as means ± standard deviation (SD). For statistical analyses Graphpad Prism software (version 8.3.0 from GraphPad Software) was used. Shapiro–Wilk test was used to determine the normality of data distribution. Unpaired two-tailed *t*-test was used to calculate statistical differences between two groups. One-way analysis of variance (ANOVA) with post-hoc Tukey’s or Dunnett’s multiple comparisons tests was used for statistical comparisons between three or more groups. A P-value of less than 0.05 was considered statistically significant. Each experiment was performed in technical duplicates or triplicates. Differences in the survival rate were calculated using Kaplan–Meier plots and analyzed with log-rank test. Information on the group size, statistical analysis used as well as P-values is provided in the figure captions.

### Ethics approval and consent to participate

The study with human bone marrow was approved by Local Bioethical Committee at the Medical University of Warsaw (approval number KB/155/2018). Ethics approval for animal studies was provided by the 1st Local Ethics Committee in Warsaw (approval No. 618/2018), and in accordance with the requirements of the EU (Directive 2010/63/EU) and Polish (Dz. U. poz. 266/15.01.2015) legislation. The study is reported in accordance with ARRIVE guidelines.

## Results

### MM progression is associated with ARG1 induction in myeloid cells

**Figure 1:**
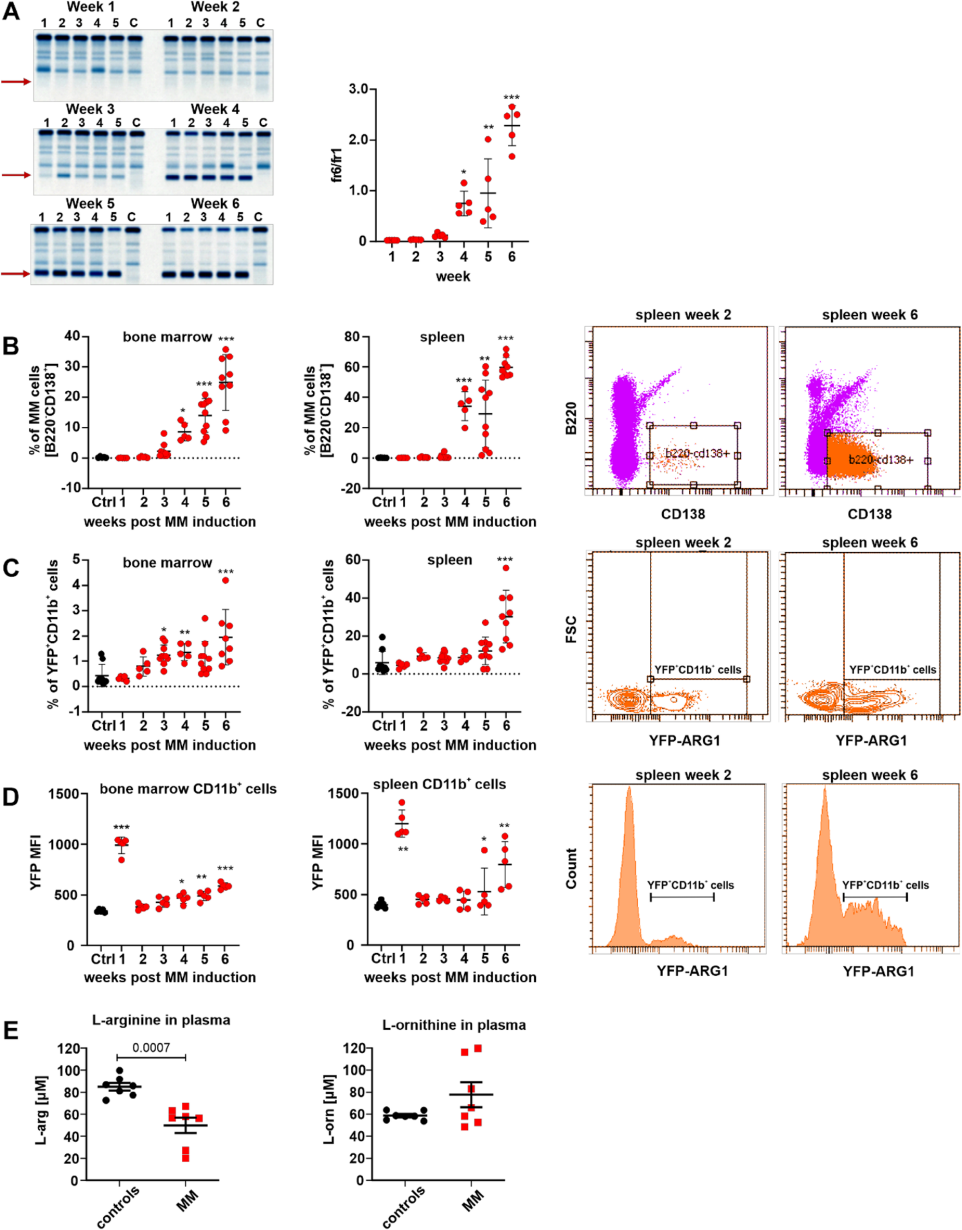
Vĸ*MYC MM progression in mice is associated with increased expression of ARG1-expressing myeloid cells in the bone marrow and spleen and decreased serum ʟ-arg concentration. YARG mice were inoculated i.v. with 1 × 10^6^ of Vĸ*MYC cells. (**A**) Increase in the monoclonal protein fraction in serum protein electrophoresis (left, red arrows) and fraction 6 (monoclonal protein)-to-fraction 1 (albumins) ratio (right) in time; C—control YARG mouse not inoculated with Vĸ*MYC cells. *P = 0.0224; **P = 0.0013, ***P < 0.0001 vs week 1; one-way ANOVA with Dunnett’s post-hoc test. (**B**) Percentage of MM cells (live CD45^+^B220^-^CD138^+^) in the bone marrow (left) and spleen (middle left) of Vĸ*MYC-bearing mice in time evaluated in flow cytometry, ctrl—YARG mice not inoculated with Vĸ*MYC cells. *P = 0.0485; **P = 0.0001, ***P < 0.0001 vs ctrl; one-way ANOVA with Dunnett’s post-hoc test. Representative spleen dot plots: week 2 (middle right) and week 6 (right). (**C**) Percentage of ARG1-expressing myeloid cells (YFP^+^CD11b^+^) in the bone marrow (left) and spleen (middle left) of Vĸ*MYC-bearing mice in time, ctrl—YARG mice not inoculated with Vĸ*MYC cells. *P = 0.0202; **P = 0.0343, ***P < 0.0001 vs ctrl; one-way ANOVA with Dunnett’s post-hoc test. Representative spleen dot plots: week 2 (middle right) and week 6 (right). (**D**) Mean fluorescence intensity (MFI) of YFP (ARG1) in the myeloid cells (live CD45^+^CD11b^+^) in the bone marrow (left) and spleen (right) of Vĸ*MYC-bearing mice in time, ctrl—YARG mice not inoculated with Vĸ*MYC cells. (left): *P = 0.0014; **P = 0.0002, ***P < 0.0001 vs ctrl; (middle left): *P = 0.0011; **P < 0.0001 vs ctrl; one-way ANOVA with Dunnett’s post-hoc test. Representative spleen histograms: week 2 (middle right) and week 6 (right). (**E**) Serum concentration of ʟ-arginine (left) and ʟ-ornithine (right) in Vĸ*MYC-bearing mice in the 6th week of MM development, ctrl—YARG mice not inoculated with Vĸ*MYC cells; P-value was calculated with unpaired two-tailed *t*-test. All graphs present means ± SD, n = 5–9.

**Figure 2:**
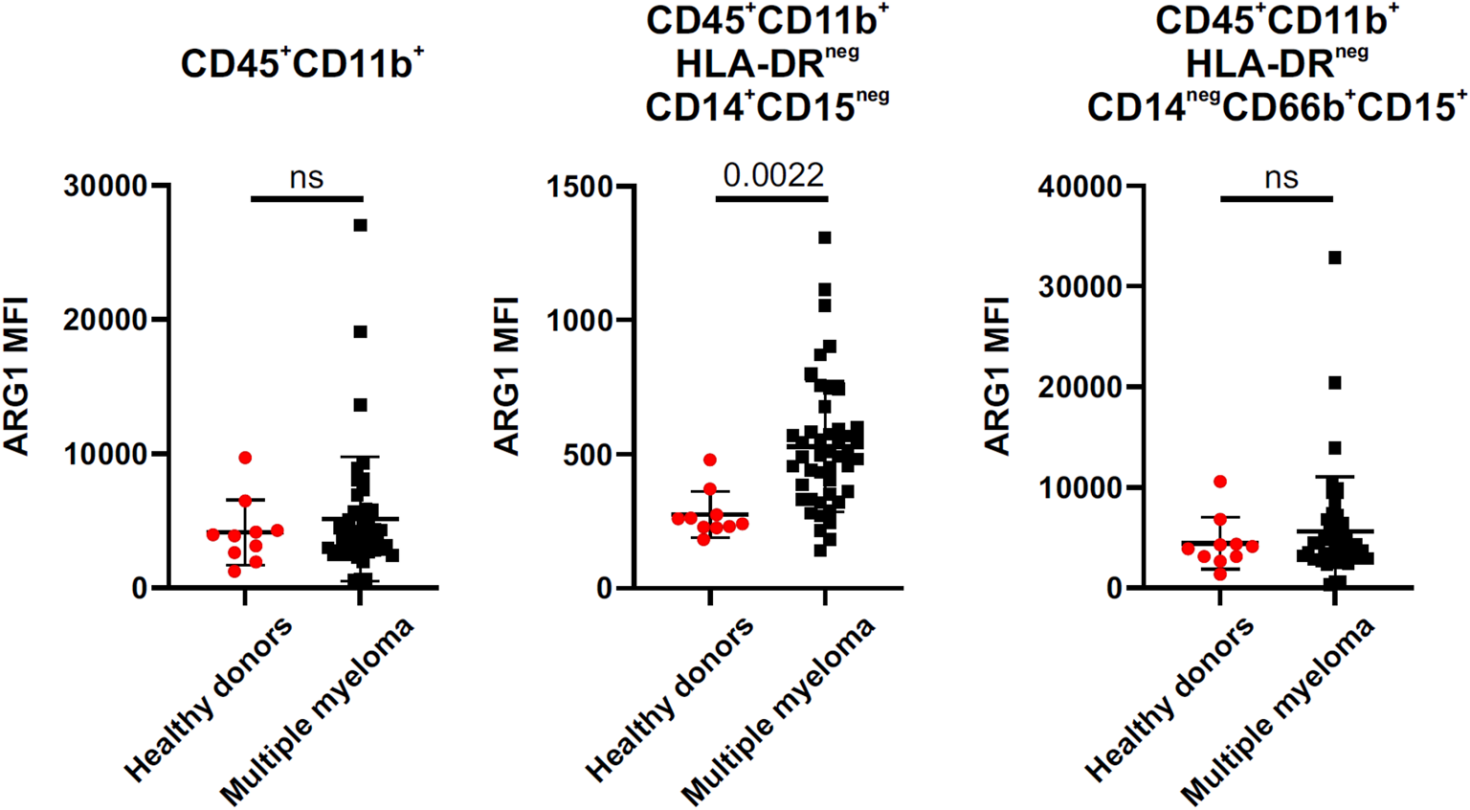
ARG1 levels are increased in the bone marrow monocytic cells in MM patients. The intensity of the intracellular staining for ARG1 in the myeloid cells subsets in the bone marrow of healthy individuals and MM patients evaluated in flow cytometry. General myeloid cells were identified as living CD45^+^CD11b^+^ (left), monocytic cells—as living CD45^+^CD11b^+^HLA-DR^neg^CD14^+^CD15^neg^ (middle), and granulocytic cells—as living CD45^+^CD11b^+^HLA-DR^neg^CD14^neg^CD66b^+^CD15^+^ (right). P-value was calculated with an unpaired two-tailed *t*-test. All graphs present means ± SD, n = 10 (healthy), n = 46 (MM).

Within the tumor microenvironment ARG1 is produced by myeloid-lineage cells, mainly immature granulocytic and monocytic myeloid-derived suppressor cells (G-MDSCs and M-MDSCs, respectively) as well as mature, M2-type macrophages, and neutrophils^9^. It has not been investigated so far whether and to what extent myeloid ARG1 can regulate tumor progression in multiple myeloma. To address this issue we have used a murine model of MM, syngeneic with C57BL/6 mice. In this model, mice are inoculated intravenously (i.v.) with cells isolated from the spleens of diseased transgenic Vκ*MYC mice^20^. MM cells (referred to as Vκ*MYC cells) are routinely propagated by transplantation into C57BL/6 mice. We observed that inoculation of 1 × 10^6^ Vκ*MYC cells into transgenic B6.129S4-Arg1^*tm1Lky*^/J mice (hereafter referred to as YARG mice) leads to a progressive increase in the serum monoclonal protein levels as well as the number of B220^-^CD138^+^ MM cells in the bone marrow and the spleens (Fig. 1A, B). YARG mice co-express yellow fluorescent protein (YFP) and ARG1 under the same ARG1 promoter^25^. Tumor progression was accompanied by the increased percentage of YFP^+^CD45^+^CD11b^+^ myeloid cells, i.e. producing ARG1, both in the bone marrow (Fig. 1C left) and the spleens (Fig. 1C right). A time-course analysis of YFP expression revealed that 1 week after inoculation of MM cells there is a significant increase in ARG1 levels in myeloid cells that is restored to control levels within the next week (Fig. 1D). Then, starting from week 4, the ARG1 levels begin to rise again with disease progression and achieve the highest levels in advanced disease. A detailed analysis of myeloid cells population revealed that both in the bone marrow and in the spleens Ly6C^+^ and myeloid dendritic cells up-regulate ARG1 with disease progression, but in Ly6G^+^ cells ARG1 is maintained on the same level throughout the observation period (with the exception of the first week) (Suppl. Figs. 7 and 8). Moreover, significantly lower ʟ-arginine concentrations were measured in the plasma of Vκ*MYC-bearing mice as compared with controls (Fig. 1E). Analysis of BM myeloid cells from healthy donors and MM patients also revealed that ARG1 levels are increased in monocytic CD45^+^CD11b^+^HLA-DR^neg^CD14^+^CD15^neg^ cells (Fig. 2).

### Myeloid cells impair T-cell proliferation

**Figure 3:**
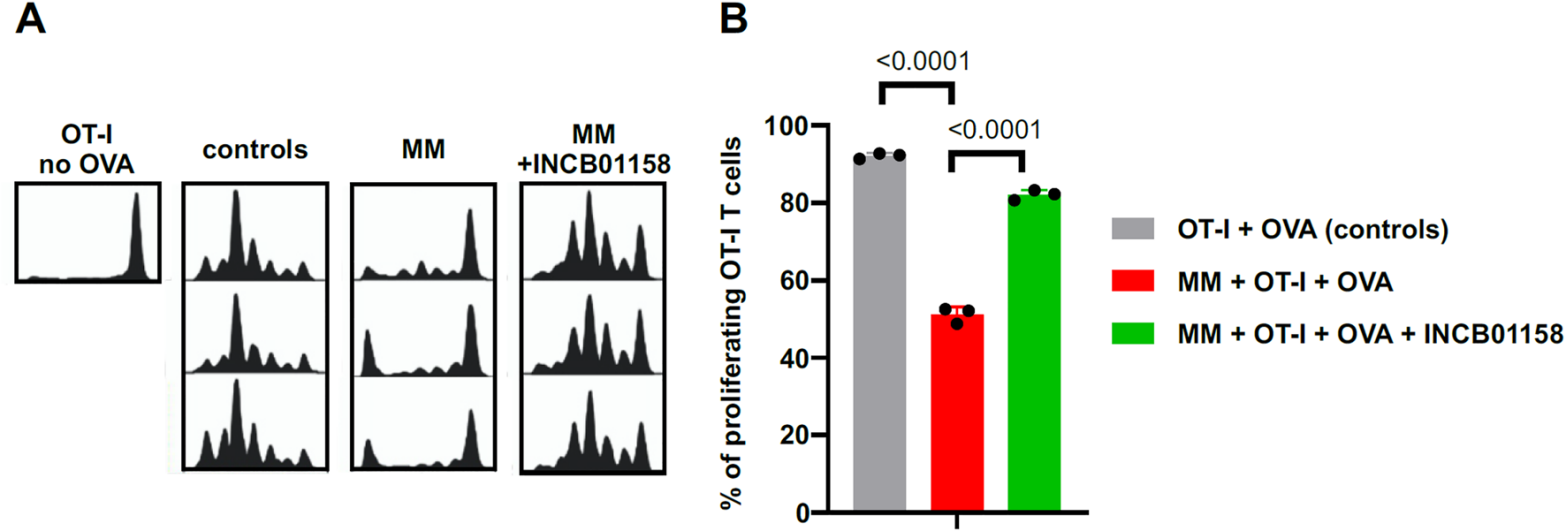
MM impairs proliferation of murine antigen-specific T cells in an ARG-dependent manner. C57BL/6 mice were i.v. inoculated with 1 × 10^6^ of Vĸ*MYC cells. In the 6th week of MM development, the mice were transferred with 1.5–2 × 10^6^ of CTV-stained CD8^+^ OT-I T cells, followed by the i.v. immunization with 10 µg of OVA protein on the next day. Proliferation of OT-I T cells in the spleens was evaluated in flow cytometry 3 days post immunization. ARG inhibitor INCB01158 was administered twice daily beginning from week 3 post MM induction. (**A**) Proliferation histograms. (**B**) Percentage of proliferating OT-I T cells in control (healthy C57BL/6 mice transferred with OT-I T cells and immunized with OVA protein) and MM-bearing mice treated were indicated with ARG inhibitor INCB01158. Graph presents means ± SD, n = 3. P-value was calculated using one-way ANOVA with Tukey’s post-hoc test.

**Figure 4:**
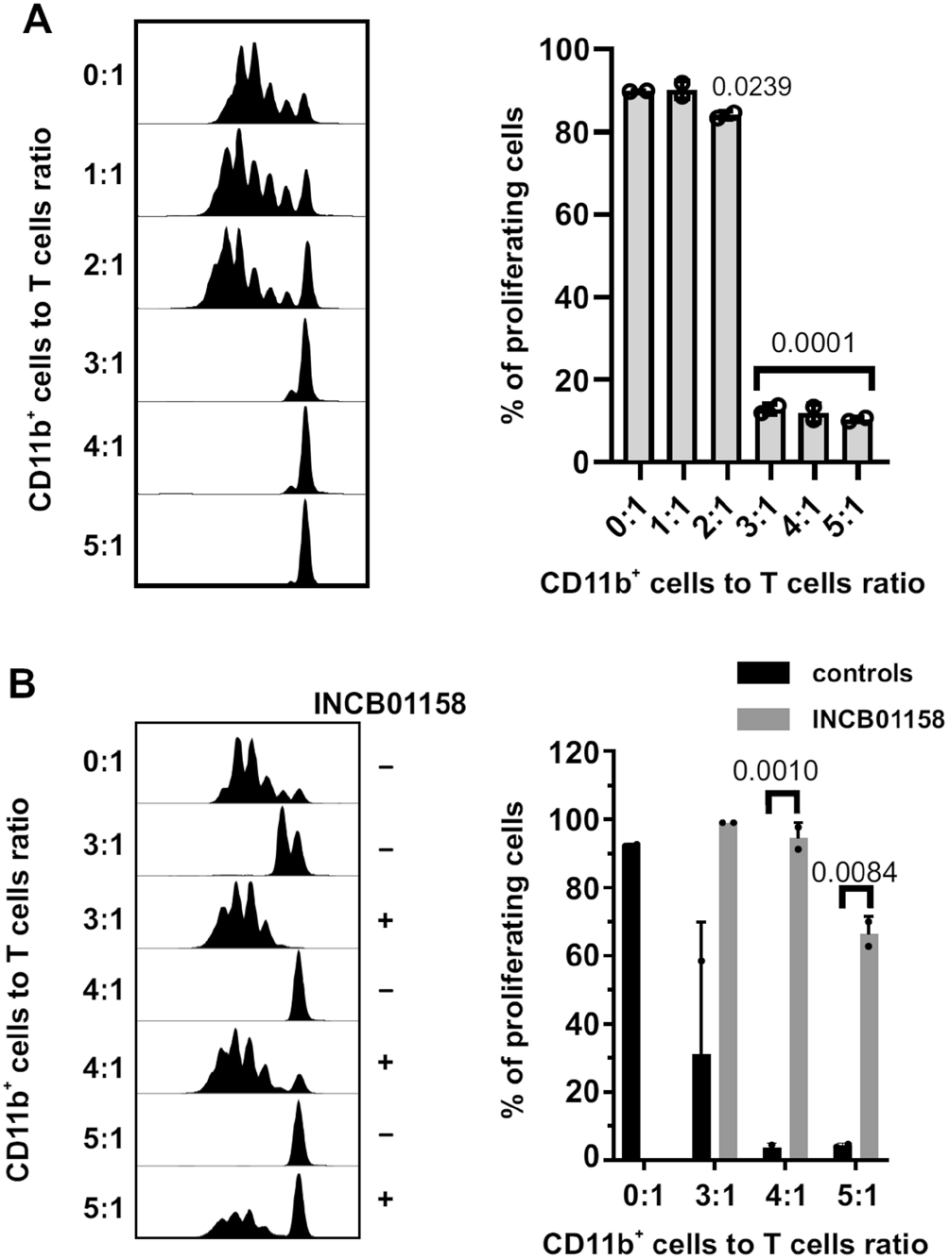
Murine MM-associated myeloid cells impair T cells proliferation in an ARG-dependent manner. Splenic myeloid cells inhibit CD8^+^ T cells proliferation ex vivo. CD11b^+^ cells were immunomagnetically isolated from the spleens of healthy or Vĸ*MYC-bearing C57BL/6 mice and ex vivo co-cultured with CTV-stained anti-CD3/CD28 beads-activated CD8^+^ T cells from healthy C57BL/6 mice at the indicated ratios. (**A**) Proliferation histograms (left) and percentages of proliferating CD8^+^ T cells (right) evaluated in flow cytometry. Graph presents mean ± SD, n = 2. P-value was calculated with one-way ANOVA with Dunnett’s post-hoc test [experimental group vs control (0:1 CD11b^+^ cells to T cells ratio)]. (**B**) ARG inhibitor restores CD11b^+^ cells-dependent T cells proliferation. Experiment was setup as described above. Where indicated, ARG inhibitor INCB01158 was added at 1 µM concentration for 72-h co-incubation time. Proliferation histograms (left) and percentages of proliferating CD8^+^ T cells (right). Graph presents means ± SD, n = 2. P-value was calculated with one-way ANOVA with Tukey’s post-hoc test (at the indicated ratio: 1 µM INCB01158 vs no ARG inhibitor group).

To see whether a drop in ʟ-arginine concentration associated with tumor progression leads to impaired T-cell proliferation, we have adoptively transferred OT-I cells into control and Vκ*MYC-bearing mice and challenged mice with OVA. A significant inhibition of antigen-induced T-cell proliferation was observed in mice with MM as compared with controls (Fig. 3). Notably, administration of ARG1 inhibitor (INCB01158) together with OT-I cells has nearly completely prevented inhibition of T-cell proliferation in MM-bearing mice, indicating that increased ʟ-arginine degradation plays an important role in systemic inhibition of antigen-induced T-cell proliferation. Similarly, co-culture of CD11b^+^ myeloid cells isolated from Vκ*MYC-bearing mice inhibited proliferation of T-cells triggered by anti-CD3/CD28 microbeads at myeloid-to-T-cell ratios higher than 3:1 (Fig. 4A). ARG1 inhibitor has restored T-cell proliferation in this setting (Fig. 4B).

**Figure 5:**
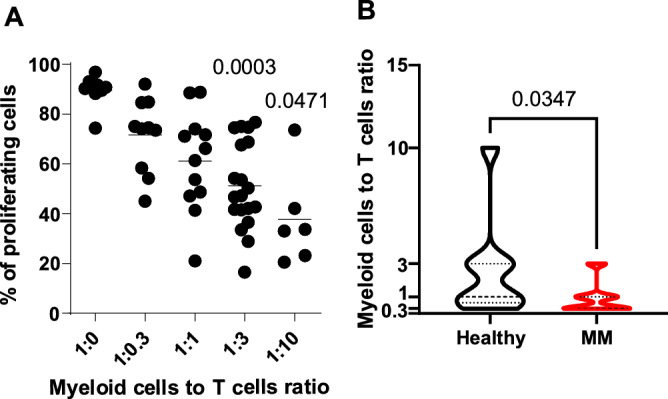
Human MM-associated bone marrow myeloid cells impair T cells proliferation. (**A**) Myeloid cells were isolated from the bone marrow of MM patients and co-cultured with CTV-stained anti-CD3/CD28 beads-activated CD4^+^ T cells from healthy blood donors at the indicated ratios. Percentages of proliferating CD4^+^ T cells evaluated in flow cytometry; bars present means, n = 6–20. P-value was calculated with mixed-effect analysis with Dunnett’s post-hoc test vs 0:1 myeloid cells to T cells ratio. (**B**) MM bone marrow-derived myeloid cells inhibit T cells proliferation at lower ratios than healthy bone marrow-derived myeloid cells. Myeloid cells derived from the bone marrow of healthy individuals (n = 10) and MM patients (n = 20) were co-cultured with CTV-stained anti-CD3/CD28 beads-activated CD4^+^ T cells from healthy blood donors at indicated ratios. Violin plots, P-value was calculated with unpaired two-tailed *t*-test.

To see whether myeloid cells from bone marrow of patients with MM can suppress the proliferation of T-cells, we have isolated myeloid cells from bone marrow aspirates of 20 MM patients and incubated these cells at various ratios with anti-CD3/CD28 stimulated CD4^+^ cells. Myeloid cells inhibited T-cell proliferation in a ratio-dependent manner (Fig. 5A). We have then compared the suppressive potential of myeloid cells isolated from BM of healthy donors (n = 10) and MM patients (n = 20), and observed that T-cell proliferation is inhibited at lower ratios of myeloid cells, when these cells were isolated from patients with MM (Fig. 5B).

### MM progression is impaired in mice with ARG1-deficient myeloid cells

**Figure 6:**
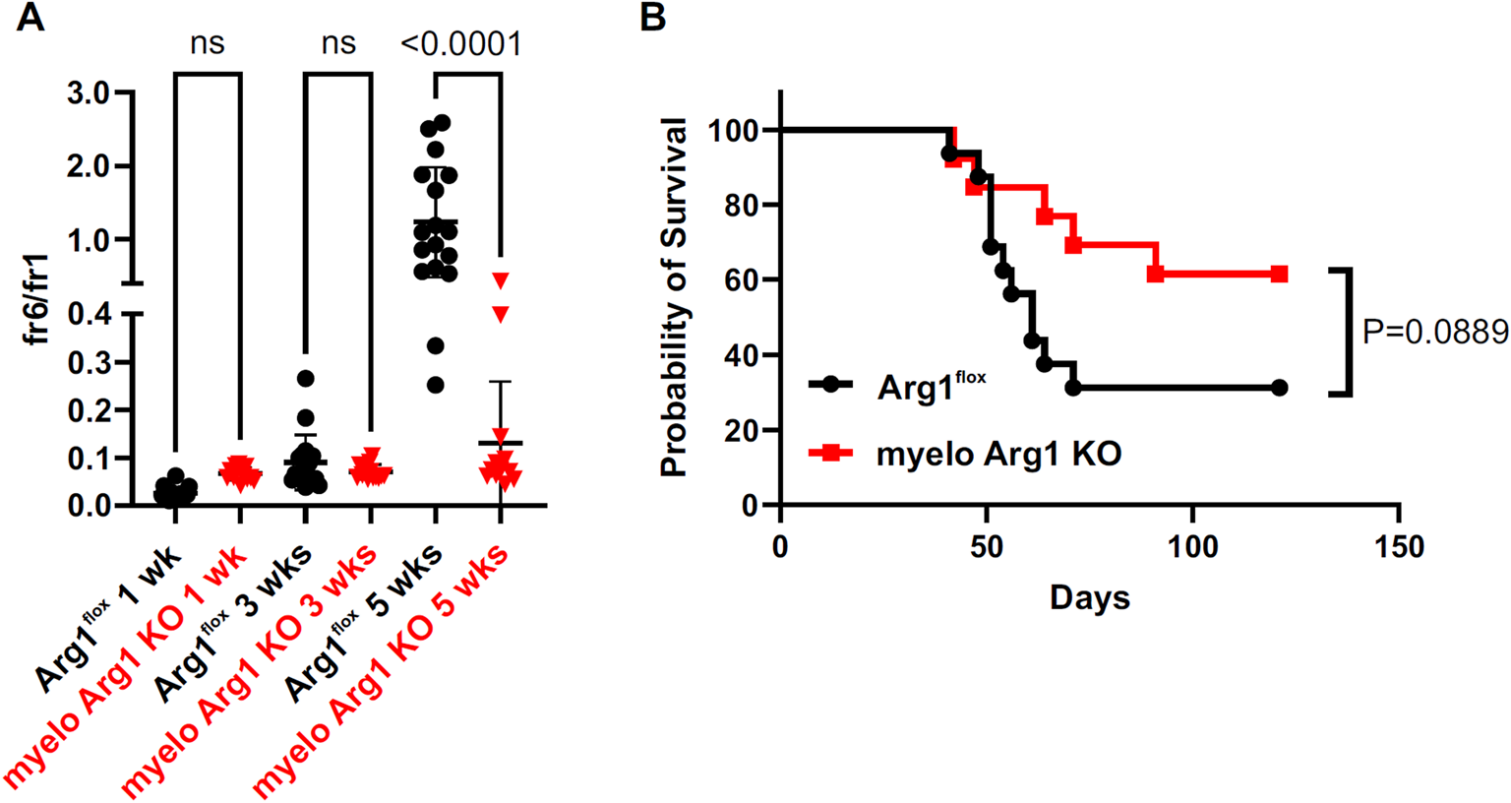
Vĸ*MYC MM progression is impaired in mice lacking functional ARG1 in myeloid cells. Arg1^flox^ (n = 17) and myelo Arg1 KO mice (n = 13) were inoculated i.v. with 1 × 10^6^ of Vĸ*MYC cells. (**A**) Fraction 6 (monoclonal protein)-to-fraction 1 (albumins) ratio in time (1, 3 and 5 weeks after MM cells inoculation); means ± SD. P-values were calculated with one-way ANOVA with Dunnett’s post-hoc test. (**B**) Animal survival curve. P-value was calculated with log-rank test.

**Figure 7:**
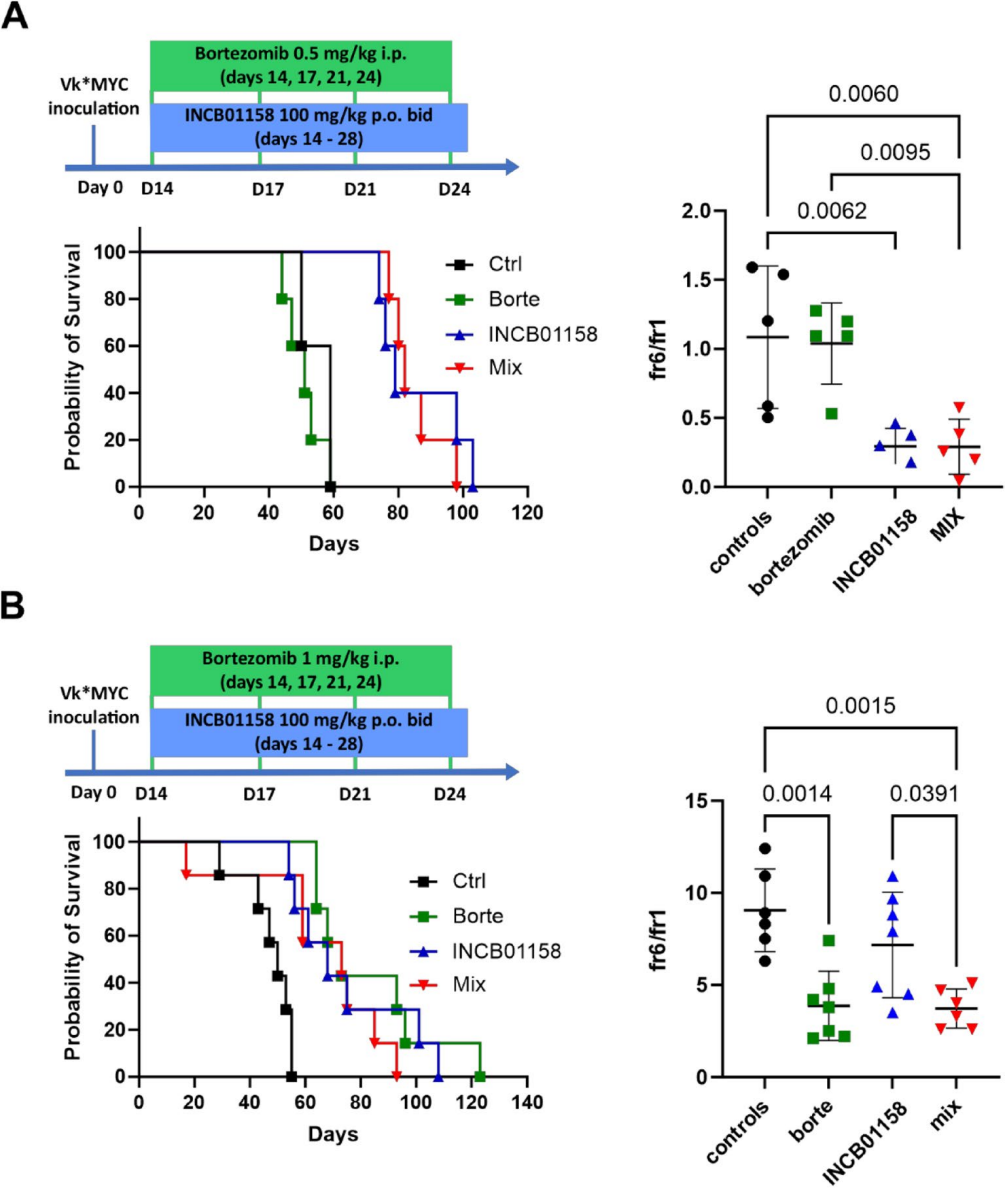
ARG inhibitor does not improve anti-myeloma effects of bortezomib. C57BL/6 mice were inoculated i.v. with 1 × 10^6^ of Vĸ*MYC cells and were treated with bortezomib [0.5 mg/kg (**A**) or 1 mg/kg (**B**) i.p.) and ARG inhibitor INCB01158 (100 mg/kg p.o.) at indicated regimens (top left). Bottom left: animal survival curves. Right: fraction 6 (monoclonal protein)-to-fraction 1 (albumins) ratio 24 h. post treatment; means ± SD. P-values were calculated with one-way ANOVA with Tukey’s post-hoc test, n = 5–8.

To determine the role of myeloid ARG1 in MM progression we have crossed Arg1^*flox*^ mice with LysMcre mice to obtain animals that have no ARG1 in myeloid lineage cells (myelo Arg1 KO mice). We observed that Vκ*MYC progression measured by monoclonal protein fraction (fr6) to albumins fraction (fr1) ratio (Fig. 6A) is slower in these animals and their survival is prolonged as compared with Arg1^flox^ mice (Fig. 6B). Therefore, to see whether targeting arginase activity might be relevant in the treatment of MM we treated Vκ*MYC-bearing mice with arginase inhibitor either alone or in combination with bortezomib, a backbone of most MM therapeutic regimens. Several different therapeutic regimens have been tested to see whether arginase inhibition might improve therapeutic outcomes of bortezomib, including subtherapeutic doses of proteasome inhibitor. In none of the therapeutic settings arginase inhibitors could improve antitumor effects of bortezomib (Fig. 7).

### Arginase inhibition reduces bortezomib-induced cardiotoxicity

**Figure 8:**
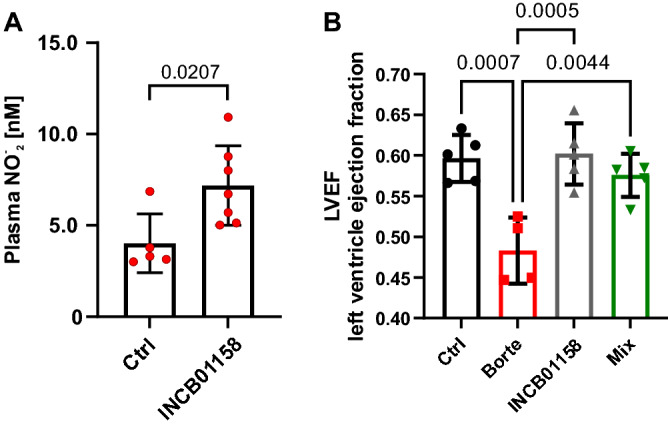
ARG inhibitors reduce bortezomib-induced cardiotoxicity. (**A**) Plasma nitrite (NO_2_^-^) concentrations in C57BL/6 mice treated with 100 mg/kg i.p. INCB01158 were measured on day 7. Data show means ± SD; n = 5–7; P-values vs ctrl were calculated with one-way ANOVA with Dunnett’s post hoc test, n = 5. (**B**) Left ventricle ejection fractions (LVEF) measured with echocardiography in C57BL/6 mice on day 7 of bortezomib (1 mg/kg i.p.) and/or INCB01158 (100 mg/kg p.o. twice daily) treatment; graphs present means ± SD; n = 4–5; P-values were calculated with one-way ANOVA with Tukey’s post-hoc test.

The use of proteasome inhibitors is associated with a significant incidence of cardiovascular adverse events^26–28^. We have tested a library of compounds that were previously reported to exert direct cytoprotective effects in cardiomyocytes, including cyclooxygenase inhibitors, erythropoietin, ceftriaxone, guanabenz, statins, PPARγ agonists, 17-AAG, or vorinostat. None of those compounds could diminish cardiotoxic effects of bortezomib in cell culture experiments (Supplementary Fig. 10A). Sildenafil, however, significantly improved left ventricle ejection fraction in bortezomib-treated rats (Supplementary Fig. 10B). Considering that sildenafil is a phosphodiesterase-5 inhibitor that reduces the metabolism of cGMP we hypothesized that arginase inhibition, which leads to increased ʟ-arginine concentration in the serum, might increase substrate availability for nitric oxide (NO) synthesis. NO activates soluble and membrane-bound guanylate cyclases, which synthesize cyclic guanylate monophosphate (cGMP), subsequently activating cGMP-kinase. Administration of INCB01158 at 100 mg/kg to Vk*MYC-bearing mice led to significantly increased plasma NO_2_^-^ concentrations (Fig. 8A). Treatment of mice with bortezomib at a dose of 1 mg/kg led to a significant drop in the left ventricle ejection fraction (LVEF). This effect was completely prevented by concomitant administration of INCB01158 arginase inhibitor (Fig. 8B).

## Discussion

In MM, malignant cells engage in bi-directional interactions with BM microenvironment through direct contact or cytokine production. For example, MM cells can induce expansion and survival of immature and mature myeloid cells^29^, whereas the latter promote proliferation of tumor cells and are involved in the development of immune suppression^16,30,31^. In newly diagnosed MM patients, increased percentages of CD14^+^ monocytic-MDSCs have been found in peripheral blood, when compared with healthy controls. In other studies, mostly CD15^+^ granulocytic-MDSCs have been reported at increased frequencies in peripheral blood of MM patients^16,32^. Both MDSCs subsets exert suppressive activity against autologous T-cells^33,34^. Moreover, a recent study of 267 newly diagnosed MM patients revealed that the frequency of mature neutrophils, but no other granulocytic subset, negatively correlates with patients outcome^35^. Analysis of gene expression profiles revealed that among the most up-regulated genes in mature neutrophils from MM patients is ARG1 and these cells turned out to have a defective phagocytic activity and exert immunosuppressive functions due to ARG1 over-expression^36^. Studies in Vκ*MYC model in mice revealed that IL-18 increases immunosuppressive activity of PMN- and M-MDSCs that have up-regulated ARG1 levels^37^. Using transgenic reporter mice we measured the levels and analyzed the phenotypes of myeloid cells expressing ARG1 upon inoculation of Vκ*MYC cells. In all animals we observed that within a week of inoculation of tumor cells there is a robust increase in both the numbers of YFP^+^ cells as well as the intensity of YFP fluorescence, indicating that nonspecific inflammation, possibly associated with inoculation of tumor cells and/or their cell death, leads to expansion of cells producing increased amounts of ARG1. Within the next week, ARG1 returned to the levels observed in control mice, and then starting from week 4 after inoculation of Vκ*MYC cells, both the number of ARG1^+^ cells as well as the levels of ARG1 gradually increased, correlating with tumor progression (Fig. 1 and Suppl. Figs. 8–9).

Myeloid cells obtained from the BM of MM patients were previously reported to inhibit T-cell proliferation, which was partially restored by pharmacologic arginase inhibition^16,36^. A recent study indicated that mature neutrophils isolated from MM patients can induce strong proliferation of even anergic T-cells, when arginase-mediated ʟ-arginine metabolism is inhibited^38^. Our results show that arginase inhibitor nearly completely restores T-cell proliferation inhibited by myeloid cells isolated from Vκ*MYC-bearing mice (Fig. 4B), indicating that the suppressive effects of myeloid cells isolated from MM patients might include additional mechanisms beyond increased ARG1 activity.

To see whether ARG1 might be involved in MM progression we have inoculated Vκ*MYC cells into mice with constitutive ARG1 depletion in the myeloid lineage driven by Cre recombinase under the control of *Lyz2* promoter. The results of these experiments indicate slower tumor progression and prolonged animal survival when myeloid cells lack ARG1 (Fig. 6). Thus, we sought to investigate whether pharmacological arginase inhibition could also exert antitumor effects or would potentiate antimyeloma activity of proteasome inhibitor. INCB01158 has been previously demonstrated to exert antitumor effects in various tumor models^39^. In Vκ*MYC model we observed a significant prolongation of mice survival by INCB01158. No potentiated antitumor effects were observed in mice treated with the combination of INCB01158 and bortezomib used in suboptimal (0.5 mg/kg) or optimal (1.0 mg/kg) dose (Fig. 7A and B).

Previous studies as well as our observations reported here (Fig. 4A) indicate that ARG1-mediated arginine depletion is associated with suppression of T-cell compartment. However, further studies are required to establish whether there is a direct association between antitumor effects of ARG1 inhibition and restoration of adaptive antitumor immunity.

Cardiotoxicity, often defined as a > 10% decrease in a left ventricular ejection fraction (LVEF), is a recognized adverse effect of multiple antitumor therapeutics. Cardiovascular adverse events (CVAE) are common during proteasome inhibitor treatment and were reported to occur in 51% of patients treated with carfilzomib and 17% of those treated with bortezomib^40^. Considering that drug-induced CVAE may compromise oncologic care due to interruption of tumor treatment, it is important to identify effective monitoring strategies as well as therapeutic approaches to mitigate cardiotoxicity. With this in mind, we explored potential cardioprotective effects of arginase inhibitors in mice treated with bortezomib. As reported in previous studies bortezomib administration was associated with a significant decrease in LVEF. Concurrent treatment with INCB01158 has completely restored cardiac function in mice. The molecular mechanisms of cardioprotective effects of arginase inhibitors are yet to be experimentally addressed. However, a vast literature supports several potential explanations. Increased arginase activity was shown to reduce the availability of ʟ-arginine for nitric oxide synthase, thus reducing NO production, increasing formation of reactive oxygen species, and leading ultimately to endothelial dysfunction^41^. Arginase inhibition was shown to augment NO production and to facilitate left ventricular systolic function in doxorubicin-induced cardiomyopathy in mice^42^. Moreover, arginase inhibitors improved microvascular endothelial function in patients with type 2 diabetes^43^ and ameliorated endothelial dysfunction associated with vascular aging^44^.

Altogether, we investigated the role of ARG1 in the progression of MM in a mouse Vκ*MYC model to see whether this enzyme might be a druggable target for the treatment of MM either alone or in combination with proteasome inhibitor. To verify the concept we have carried out both genetic and drug-based approaches and shown that ARG1 is induced in myeloid cells during MM progression. Knocking-out ARG1 in myeloid cells is associated with slower tumor progression and improved mice survival. Similarly, treatment with arginase inhibitor demonstrated significant antitumor effects, but was ineffective in potentiating antitumor effects of bortezomib. However, arginase inhibitor improved LVEF that was decreased by bortezomib, indicating that targeting ARG1 might be effective in ameliorating cardiotoxicity of proteasome inhibitors. We expect that the results of our studies might contribute to improved safety of tumor treatment in at least some subgroups of cancer patients.

## Supplementary Information

Supplementary Information.

## Data Availability

The results described in the manuscript, including all relevant raw data, will be freely available from the corresponding authors to any scientist wishing to use them for non-commercial purposes.
