# Supplementary material for: Targeting arginase-1 exerts antitumor effects in multiple myeloma and mitigates bortezomib-induced cardiotoxicity
Source: Sci Rep. 2022 Nov 16;12:19660. doi: 10.1038/s41598-022-24137-1 (PMC9668840; doi:10.1038/s41598-022-24137-1)
Supplement: Supplementary file 1 — Supplementary Information. [file 41598_2022_24137_MOESM1_ESM.docx]

**Supplementary data**

**Inhibition of arginase attenuates bortezomib-induced cardiotoxicity but does not potentiate its antitumor effects in multiple myeloma**

Kavita Ramji^1^, Tomasz M. Grzywa^1,2^, Anna Sosnowska^1^, Aleksandra Paterek^3^, Marta Okninska^3^, Zofia Pilch^1^, Joanna Barankiewicz^4^, Filip Garbicz^5,6^, Katarzyna Borg^7^, Urszula Bany-Laszewicz^7^, Abdesamad Zerrouqi^1^, Beata Pyrzynska^1^, Anna Rodziewicz-Lurzynska^8^, Diana Papiernik^9^, Piotr Sklepkiewicz^9^, Hanna Kedzierska^9^, Adam Staruch^1^, Radoslaw Sadowski^1^, Olga Ciepiela^10^, Ewa Lech-Maranda^4^, Przemyslaw Juszczynski^5^, Urszula Mackiewicz^3^, Michal Maczewski^3^, Dominika Nowis^1,2*#^, Jakub Golab^1,11*#^

^1^ Department of Immunology, Medical University of Warsaw, 5 Nielubowicza Str., 02-097 Warsaw, Poland

^2^ Laboratory of Experimental Medicine, Medical University of Warsaw, 5 Nielubowicza Str., 02-097 Warsaw, Poland

^3^ Department of Clinical Physiology, Centre of Postgraduate Medical Education, 99/103 Marymoncka Str., 01-813 Warsaw, Poland

^4^ Department of Hematology, Institute of Hematology and Transfusion Medicine, 14 Indiry Gandhi Str., 02-776 Warsaw, Poland

^5^ Department of Experimental Hematology, Institute of Hematology and Transfusion Medicine, 14 Indiry Gandhi Str., 02-776 Warsaw, Poland

^6^ Postgraduate School of Molecular Medicine, Medical University of Warsaw, 1B Banacha Str., 02-097 Warsaw, Poland

^7^ Department of Diagnostic Hematology, Institute of Hematology and Transfusion Medicine, 14 Indiry Gandhi Str., 02-776 Warsaw, Poland

^8^ Central Laboratory, University Clinical Center of Medical University of Warsaw, 1A Banacha Str., 02-097 Warsaw, Poland

^9^ ExploRNA Therapeutics Ltd., 101 Zwirki i Wigury Str., 0.30, 02-089 Warsaw, Poland

^10^ Department of Laboratory Medicine, Medical University of Warsaw, 1A Banacha Str., 02-097 Warsaw, Poland

^11^ Centre of Preclinical Research, Medical University of Warsaw, 1B Banacha Str., 02-097 Warsaw, Poland.

Supplementary Table 1. Antibodies used for immunophenotyping of murine cells

| **Target** | **Fluorochrome** | **Clone** | **Dilution** | **Manufacturer** |
| --- | --- | --- | --- | --- |
| B220 | PerCp-Cy5.5 | RA3-6B2 | 1:200 | BD Biosciences |
| CD138 | APC | 300506 | 1:200 | Invitrogen |
| CD11b | VioBlue 450 | M1/70 | 1:100 | eBioscience |
| CD3e | APC | 17A2 | 1:200 | eBioscience |
| CD45.2 | V500 | 104 | 1:50 | eBioscience |
| CD8 | PerCp-Cy5.5 | 53-6.7 | 1:200 | eBioscience |
| F4/80 | PE-Cy7 | BM8 | 1:400 | eBioscience |
| Ly6C | APC | HK1.4 | 1:200 | eBioscience |
| Ly6G | PE | 1A8 | 1:200 | BioLegend |
| I-A^b^ | PerCp-Cy5.5 | M5/114.15.2 | 1:200 | eBioscience |

Supplementary Table 2. Antibodies used for immunophenotyping of human cells

| **Target** | **Fluorochrome** | **Clone** | **Dilution** | **Manufacturer** |
| --- | --- | --- | --- | --- |
| CD3ε | Alexa Fluor 488 | OKT3 | 1:100 | eBioscience |
| CD3ε | PE-Cy7 | OKT3 | 1:100 | eBioscience |
| CD4 | APC | RPA-T4 | 1:100 | BioLegend |
| CD4 | PerCP-Cy5.5 | RPA-T4 | 1:100 | eBioscience |
| CD8a | PE-Cy7 | SK1 | 1:100 | eBioscience |
| CD8a | PerCP-Cy5.5 | SK1 | 1:100 | eBioscience |
| CD11b | APC | ICRF44 | 1:200 | eBioscience |
| CD14 | PerCP-Cy5.5 | 61D3 | 1:200 | eBioscience |
| CD15 | V450 | MMA | 1:200 | eBioscience |
| CD38 | PE | HB7 | 1:100 | eBioscience |
| CD45 | V500 | HI30 | 1:50 | BD Biosciences |
| CD66b | FITC | G10F5 | 1:200 | eBioscience |
| CD68 | FITC | eBioY1/82A | 1:100 | eBioscience |
| CD247 (CD3ζ) | PE | 6B10.2 | 1:100 | eBioscience |
| ARG1 | PE | Polyclonal | 1:10 | R&DSystems |
| HLA-DR | PE-Cy7 | L243 | 1:200 | eBioscience |


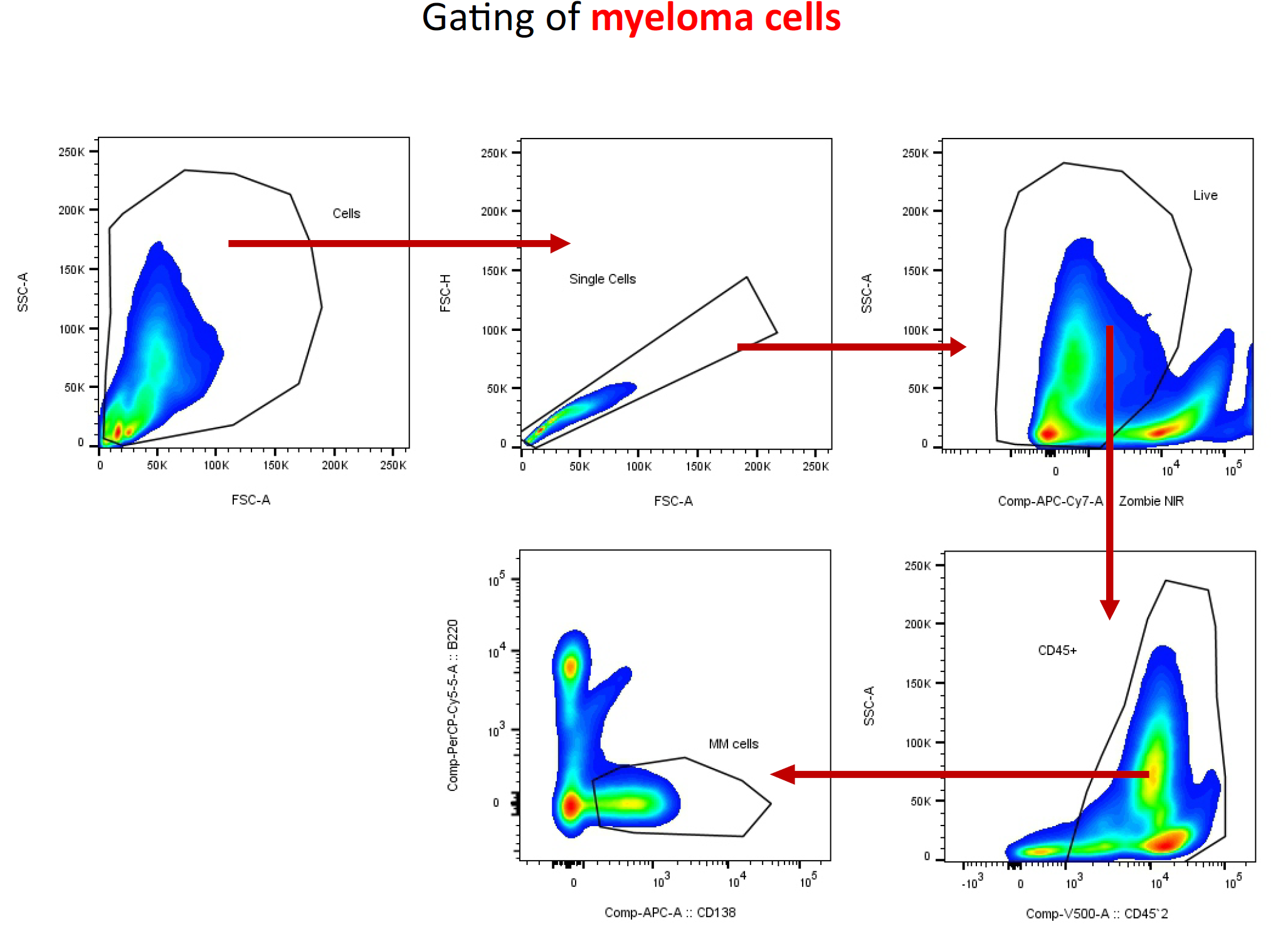


**Supplementary Fig. 1. Gating strategy used to identify MM cells in the mouse spleen and bone marrow.** A minimum of 50 000 cells were acquired within the CD45^+^ gate. Graphs were generated using FlowJo v7.6.5.

**Supplementary Fig. 2. Gating strategy used to identify major populations of myeloid cells and their ARG1 (YFP) levels in the mouse spleen and bone marrow.** A minimum of 50 000 cells were acquired within the CD45^+^ gate. Graphs were generated using FlowJo v7.6.5.

**Supplementary Fig. 3. Gating strategy used to identify macrophages and their ARG1 (YFP) levels in the mouse spleen and bone marrow.** A minimum of 50 000 cells were acquired within the CD45^+^ gate. Graphs were generated using FlowJo v7.6.5.

**Supplementary Fig. 4. Gating strategy used to identify dendritic cells (DCs) and their ARG1 (YFP) levels in the mouse spleen and bone marrow.** A minimum of 50 000 cells were acquired within the CD45^+^ gate. Graphs were generated using FlowJo v7.6.5.

**Supplementary Fig. 5. Gating strategy used to identify monocytes/monocytic MDSCs and granulocytes/granulocytic MDSCs and their ARG1 (YFP) levels in the mouse spleen and bone marrow.** A minimum of 50 000 cells were acquired within the CD45^+^ gate. Graphs were generated using FlowJo v7.6.5.

**Supplementary Fig. 6. Gating strategy used to identify subpopulations of Ly6C^+^ and Ly6G^+^ cells and their ARG1 (YFP) levels in the mouse spleen and bone marrow.** A minimum of 50 000 cells were acquired within the CD45^+^ gate. Graphs were generated using FlowJo v7.6.5.


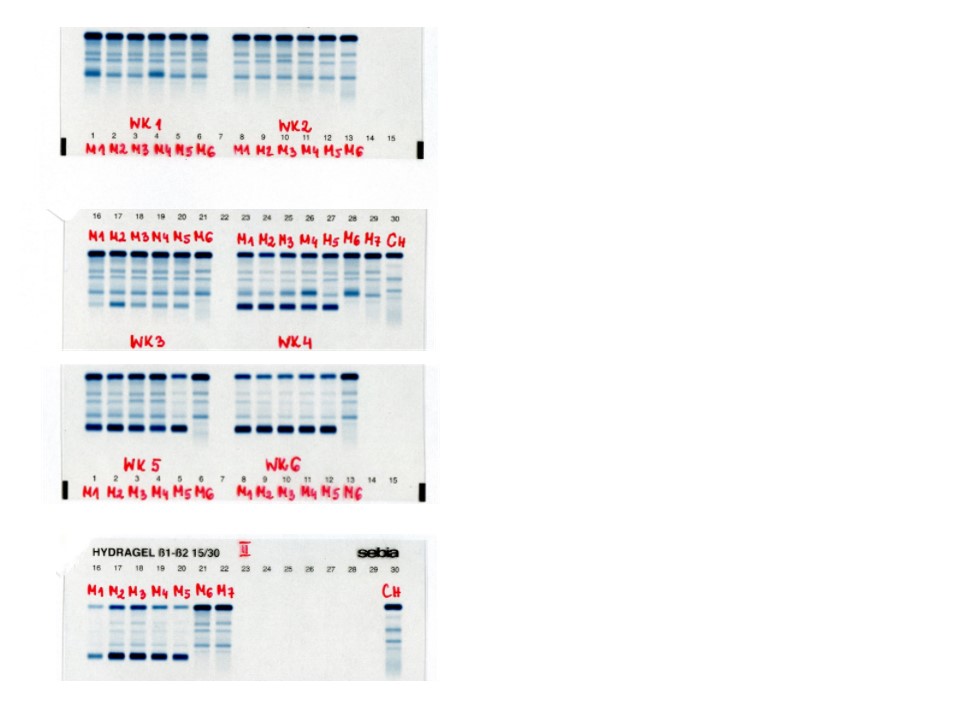


**Supplementary Fig. 7. Uncropped serum protein electrophoresis gels shown in Figure 1.**

**Supplementary Fig. 8. ARG1 (YFP) levels in the subpopulations of myeloid cells present in the spleens** **of Vĸ*MYC cells-bearing mice.** Spleens of the Vĸ*MYC cells-bearing YARG mice were harvested weekly for six consecutive weeks post inoculation of MM cells and immunophenotyped by flow cytometry. Control group consisted of 10 mice (5 euthanized on the day of the start of the experiment and 5 – on the final day of the experiment), all other groups consisted of 5 mice. A minimum of 50 000 cells were acquired within the CD45^+^ gate. Graphs were generated using FlowJo v7.6.5. Graphs present means ± SD. P-values were calculated with one-way ANOVA with Dunnett’s post hoc test.

**Supplementary Fig. 9. ARG1 (YFP) levels in the subpopulations of myeloid cells present in the bone marrow of Vĸ*MYC cells-bearing mice**. Femoral bone marrow of the Vĸ*MYC cells-bearing YARG mice was harvested weekly for six consecutive weeks post inoculation of MM cells and immunophenotyped by flow cytometry. Control group consisted of 10 mice (5 euthanized on the day of the start of the experiment and 5 – on the final day of the experiment), all other groups consisted of 5 mice. A minimum of 50 000 cells were acquired within the CD45^+^ gate. Graphs were generated using FlowJo v7.6.5. Graphs present means ± SD. P-values were calculated with one-way ANOVA with Dunnett’s post hoc test.

**Supplementary Fig. 10. Sildenafil shows protection against bortezomib-induced cardiotoxicity in vivo but not in vitro. A.** In vitro screening of potential cytoprotective agents against bortezomib-induced cytostatic/cytotoxic effects in H9c2 rat cardiac myoblast cells. H9c2 cells were seeded on 96-well plates and incubated for 48 hrs with bortezomib and compounds of known cytoprotective activity (from lop left: 17-AAG, atorvastatin, ceftriaxone, erythropoietin, guanabenz, pioglitazone, SAHA, and sildenafil), at indicated concentrations. Cytostatic/cytotoxic effects were evaluated with crystal violet staining. Graphs show mean means ± SD, n=3. **B.** Cardiac left ventricle ejection fraction of male Wistar rats treated for 2 weeks with bortezomib (0.2 mg/kg i.p. three times a week) and sildenafil (0.7 mg/kg i.p. daily). Graphs show mean means ± SD, n=6-7. * P<0.05, ** P<0.01, unpaired two-tailed *t* test.

**Supplementary materials and methods**

**Cells**

Rat cardiomyoblastic (H9c2) cell line was purchased from ATCC (CRL-1446). Cells were cultured in Dulbecco’s modified Eagle’s medium supplemented with supplemented with 10% (v/v) FBS, 2 mM glutamine, 1% (v/v) penicillin/streptomycin (all from Thermo Fisher Scientific). Cells were maintained at 37°C, 5% CO_2_, in humidified atmosphere.

**Drugs and reagents**

17-(Allylamino)-17-demethoxygeldanamycin (17-AAG, Sigma-Aldrich), atorvastatin (Pfizer), ceftriaxone sodium (Merck), human recombinant erythropoietin (Peprotech), guanabenz acetate (Merck), pioglitazone hydrochloride (Merck), SAHA (N-hydroxy-N′-phenyl-octanediamide, Merck), sildenafil (Pfizer) were used.

**Cytostatic/cytotoxic assay**

The cytostatic/cytotoxic effects of tested agents combinations in H9c2 cells were measured using crystal violet staining. Briefly, the cells were dispensed into 96- well plates (Sarstedt, Numbrecht, Germany) at 3×10^3^ cells per well and allowed to attach overnight. The following day the investigated agents were added at indicated concentrations. After 48 h-incubation the cells were rinsed with PBS and stained with 0.5% crystal violet in 2% ethanol for 10 minutes at room temperature. Plates were washed four times with tap water and cells were lysed with 1% SDS solution. Absorbance was measured at 595 nm using a microplate reader (ASYS UVM 340, Biochrom, Berlin, Germany). The relative viability was calculated according to the following formula: % viability=[(Ae-Ab)/(Ac-Ab)]×100%, where Ae is the experimental absorbance, Ab is the background absorbance, and Ac is the absorbance of untreated controls.

**In vivo experiments in rats**

Male Wistar rats (350 to 400 g) were used in the experiments. Breeding pairs were obtained from the Animal House of the Medical Research Center, Polish Academy of Sciences (Warsaw, Poland). Animals were housed in controlled environmental conditions conventional animal facility of the Medical University of Warsaw with water and food provided ad libitum. The experiments were performed in accordance with the guidelines approved by the 2^nd^ Local Ethics Committee in Warsaw (approval No. 22/2007). Rats were injected i.p. with 0.2 mg/kg of bortezomib (Adamed) three times a week for 2 weeks and 0.7 mg/kg sildenafil (Pfizer) daily for 2 weeks.

**Echocardiography**

Echocardiography was performed using MyLab25 (Esaote, Italy) with a 13 MHz linear array transducer. Each rat was examined at baseline, 24 hours, 5 days, 8 days, and 15 days after treatment initialization, and 1 and 2 weeks (day 21 and day 28, respectively) after termination of treatment (wash-out). Under light anesthesia (i.p. ketamine HCl and xylazine, 75 and 3.5 mg/kg body weight, respectively,) left ventricular (LV) end-diastolic and end-systolic diameters were determined from the short-axis view at the midpapillary level and fractional shortening was calculated as (LV diastolic - LV systolic diameter)/LV diastolic diameter. LV end-diastolic and end-systolic areas were planimetered from the parasternal long-axis view. LV ejection fraction was calculated as (LV diastolic area - LV systolic area)/LV diastolic area.

**Statistical analysis**

Data are shown as means ± standard deviation (SD). Graphpad Prism 8.3.0 (GraphPad Software) was used for statistical analyses. The normality of data distribution was tested using Shapiro-Wilk test. For statistical analyses of two groups unpaired two-tailed *t*-test was used. For statistical analyses of three or more groups one-way analysis of variance (ANOVA) followed by post-hoc Tukey’s or Dunnett’s multiple comparisons tests. A P-value of less than 0.05 was considered statistically significant. Each experiment was performed in technical duplicates or triplicates. The survival rate was computed using Kaplan-Meier plots and analyzed with log-rank test. Information on the group size, statistical analysis used as well as P-values is provided in the figure captions.

**Bibliography**

1. Sosnowska A, Chlebowska-Tuz J, Matryba P, Pilch Z, Greig A, Wolny A, et al. Inhibition of arginase modulates T-cell response in the tumor microenvironment of lung carcinoma. Oncoimmunology. 2021;10(1):1956143.

2. Chesi M, Robbiani DF, Sebag M, Chng WJ, Affer M, Tiedemann R, et al. AID-dependent activation of a MYC transgene induces multiple myeloma in a conditional mouse model of post-germinal center malignancies. Cancer Cell. 2008;13(2):167-180.

3. Chesi M, Matthews GM, Garbitt VM, Palmer SE, Shortt J, Lefebure M, et al. Drug response in a genetically engineered mouse model of multiple myeloma is predictive of clinical efficacy. Blood. 2012;120(2):376-385.

4. Maczewski M, Beresewicz A. Role of nitric oxide and free radicals in cardioprotection by blocking Na+/H+ and Na+/Ca2+ exchange in rat heart. Eur J Pharmacol. 2003;461(2-3):139-147.
